# Patient and carer experience of obtaining regular prescribed medication for chronic disease in the English National Health Service: a qualitative study

**DOI:** 10.1186/1472-6963-13-192

**Published:** 2013-05-24

**Authors:** Patricia M Wilson, Neha Kataria, Elaine McNeilly

**Affiliations:** 1 Centre for Research in Primary and Community Care, University of Hertfordshire, College Lane, Hatfield AL10 9AB, UK; 2 Princess Alexandra Hospital NHS Trust, Hamstel Road, Harlow CM20 1QX, UK

**Keywords:** Chronic disease, Patient experience, Prescribed medication, Health service delivery

## Abstract

**Background:**

The increasing burden of chronic disease is recognised globally. Within the English National Health Service, patients with chronic disease comprise of half of all consultations in primary care, and 70% of inpatient bed days. The cost of prescribing long-term medications for those with physical chronic diseases is rising and there is a drive to reduce medicine wastage and costs. While current policies in England are focused on the latter, there has been little previous research on patient experience of ordering and obtaining regular medication for their chronic disease. This paper presents findings from England of a qualitative study and survey of patients and their carers’ experiences of community and primary care based services for physical chronic diseases. Although not the primary focus of the study, the results highlighted particular issues around service delivery of repeat prescriptions.

**Methods:**

We conducted 21 qualitative in-depth interviews with 30 patients and family carers’ in two Primary Care Trusts in England. Participants were receiving community based care for diabetes, respiratory, neurological or complex co-morbidities, and ranged in age from 39–92 years old. We used a broadly inductive approach to enable themes around patient experience to emerge from the data.

**Results:**

While the study sought to gain an overview of patient experience, the findings suggested that the processes associated with ordering and obtaining regular medication – the repeat prescription, was most frequently described as a recurring hassle of managing a long-term condition. Issues for patients and carers included multiple journeys to the surgery and pharmacy, lack of synchrony and dissatisfaction with the length of prescriptions.

**Conclusion:**

Much literature exists around medication waste and cost, which led to encouragement from the NHS in England to reduce dosage units to a 28-day supply. While there has been an acknowledgement that longer supplies may be suitable for people with stable chronic conditions, it appears that there is limited evidence on the impact of shorter length prescriptions on patient and carer experience, adherence and health outcomes. Recent policy documents within England also fail to address possible links between patient experience, adherence and flaws within repeat prescription service delivery.

## Background

Chronic diseases are the major challenge facing the global health economy. The World Health Organization estimates that non-communicable diseases account for more than 60% of deaths worldwide [1]. Within the United States it is predicted that five long-term conditions (cardiovascular disease, cancer, chronic respiratory disease, diabetes, mental health) will cause a cumulative output loss of US$ 47 trillion over the next twenty years [2]. Around 15 million people in England have a long-term condition [3]. The number of chronic diseases per person increases with age [4], and these patients are the most frequent users of healthcare services. In England and Wales, patients with chronic disease account for 50% of General Practitioner (GP) appointments and 70% of all inpatient bed days [5]. Currently the flow of policies is aimed at integrating services for long-term conditions and enabling patient choice and voice in service provision [6,7]. Alongside the move towards a more seamless approach to service provision, medicines management continues to play a significant role within chronic disease management. The National Health Service (NHS) in England spent £8.2 billion on prescription drugs in primary care in 2006, around a quarter of the total expenditure on primary care. Ninety-eight per cent of these drugs were prescribed by GPs [8], with 12 of the 20 most frequently prescribed medications being for chronic diseases. Drugs for cardiovascular disease are the most commonly dispensed and cost the NHS £1.9 billion in 2006 [9].

Within the English NHS, the majority of patients with chronic diseases are over the age of 65 and are prescribed drugs with no charge to the patient, with older people receiving an average of 38 items per year [8]. In England, regular medication for stable long-term conditions is normally managed via a repeat prescription process, whereby prescriptions are issued without a consultation but are regularly reviewed by the GP [10]. The use of repeat prescriptions has vastly increased over the past 30 years, reflecting changing morbidity patterns and the introduction of computerised systems. Recent figures indicate that 80% of all prescription items dispensed in primary care are through repeat prescriptions [11], with repeat prescribing increasing with the age of the patient [12,13]. While there is general guidance for good practice in repeat prescribing within the NHS [14] several models exist for obtaining repeat prescriptions including patients submitting requests in person, by post, via email or the telephone, with GP practices often adopting a variety of methods [10]. There have been some attempts at providing an alternative model to the repeat prescription process in England. Introduced in 2005, repeat dispensing enables community pharmacists to dispense regular medicines to patients on each occasion a repeat is needed, without requiring another prescription from their GP. The GP can sign for up to 12 months of repeat prescription forms, lasting 28 days each time [15-17]. Despite some evidence that this provides a more flexible approach for patients with fewer trips to the GP practice [18] it also commits them to return to the same pharmacist every month giving rise to potential problems if a particular drug is out of stock [16,19]. In reality, repeat dispensing schemes are relatively rare with the repeat prescription the most common approach in long term conditions [8].

Internationally, there has been considerable interest in the health outcomes implications of non-adherence to prescribed medication in chronic conditions [20], and costs associated with unused medications [21-24]. However, the preoccupation with the link between non-adherence and drug wastage has overshadowed exploration of patient experience around obtaining their prescription and the possible consequences for adherence. This paper reports on a study that investigated patient experience of receiving health care from long term conditions services that had “integrating activities” [25] such as pathways of care that cross hospital and community health care providers. A focus on patient and carer experience allows exploration of whole systems of care [26] and may also reveal issues that were previously hidden to service providers and researchers [27]. The aim of the paper is to present the key themes emerging from interviews with patients and carers and predominantly concerned medicine management.

## Methods

The study was conducted between 2010–2012 and had built on an earlier mapping of the evidence [28] which found that services promoting integrated and coordinated care were relatively new, with no clear evidence on a number of outcomes including patient experience. Therefore, the main research question was ‘what is the impact on patient experience of long-term conditions services with integrating activities?’

In view of the dearth of evidence, a broadly inductive approach was undertaken to enable themes around patient experience to emerge from the data, rather than discarding any themes that did not fit with a preconceived framework. This paper reports findings from phase two of the study, but in total the study comprised of three phases. Phase one comprised of a web based scoping of long term conditions services within two counties in England which enabled identification of services that had “integrating activities” [25,29], for example; integrated teams or shared clinical processes such as pathways of care. We then approached the Primary Care Trusts (PCT) (the NHS organisation responsible for commissioning health services within a geographical area) within the counties and two agreed to participate in the study (equating to a total patient population of nearly 1,300,000). The PCTs served populations diverse in terms of demographics, ethnic and cultural populations, and rural/urban communities. Both PCTs provided services for diabetes, respiratory, long-term neurological conditions and older people with complex co-morbidities (Table 1). Following NHS Research Ethics (REC reference 09/H0302/1) and relevant research governance approval we invited all lead practitioners, managers and the commissioners of the services to participate. With the aim of gathering in-depth data about each service and perceptions of enablers and barriers to integrated working, we interviewed a total of 16 health professionals. Within phase two, the lead practitioners from each service distributed an invitation to a census sample of their patients over a 1 month period. In the case of the community matron (a community nurse with additional training in diagnostic skills and independent prescribing, usually adopting a case management approach) and respiratory services, this was to all patients and their family carers who were on the practitioner’s caseload at that time. For the diabetes and neurological services, the invitation was given to all patients who attended the clinic during that month. If interested in participating, the patient or carer directly contacted the research team via an expression of interest form and pre-paid envelope. The lead researcher (PW) then contacted the patient or carer, went through the information sheet and answered any questions. If still agreeable, a date was made for a face to face interview which took place in the patient’s home, with signed informed consent forms being completed before the interview commenced. Interviews were conducted by PW and EM who, as university based researchers, had no role in the care of the patient or carer. Good Clinical Practice (GCP) guidelines [30] were followed including ensuring participants’ anonymity and maintaining confidentiality. A total of 30 patients and family carers agreed to participate, and of these 18 participants were interviewed as a dyad (9 interviews) and the remaining 12 participants were interviewed individually. A breakdown of the participants is given in Table 2. While a topic guide (Table 3) was used to elicit views and experiences of service delivery and the long-term conditions, interviews followed a conversational style [31]. Interviews were recorded, transcribed and coded thematically [32], with NVivo software [33] being used to organise the data. Analysis of data was undertaken both within and between each long-term condition service, with at least 2 researchers independently cross-checking coded transcripts to enable inter-rater reliability. Interviews continued until data saturation was achieved [34]. This was established when no new codes were identified in the last batch of transcripts and there was agreement within the team that the data collection at that point was not adding anything new to the exploration of patient experience of long-term conditions services with integrating activities. To ensure rigour and to inform the phase three development of a pilot questionnaire aimed at measuring patient experience of long term conditions services, emerging themes and the research team’s understanding was checked with participants. Participants from phase two also undertook test-retesting of the questionnaire before it was piloted with a separate sample. The study methods have been presented following the RATS guidelines (http://www.biomedcentral.com/ifora/rats).

**Table 1:** Community based chronic disease services

**Complex needs case management model**	**Respiratory service**	**Neurological service**	**Diabetes service**
**Site A**	**Site A**	**Site A**	**Site A**
Community Matron model.	Led by respiratory nurse consultant with a team of nurse specialists, physiotherapists, and administration support.	Team of nurses and therapists.	Managed by a nurse consultant under a single budget with a number of diabetes nurse specialists.
Model adapted from United Health.	Medical consultant input though local and neighbouring acute hospitals.	Work with patients from diagnosis to end of life.	Provides community based clinics, education for GPs and practice nurses, structured self-management education.
Co-located with intermediate care teams.		Patients refer themselves in and out of the service as required.	
Loosely attached to GP practices.			
**Site B**	**Site B**	**Site B**	**Site B**
Integrated Community Team.	Covers all respiratory diseases and oxygen reviews.	3 specialist nurses.	1 diabetes nurse specialist and 1 Diabetes Practitioner Consultant.
One team per the three PCT localities.		22 bedded stroke and neurology rehabilitation unit.	Structured self-management programme is provided
Teams include community matron (case manager), district nurses, and therapists.	Led by a respiratory nurse consultant and team of nurse specialists and a physiotherapist.		Diabetes Nurse Specialist runs clinics in 2 GP centres.
Community matron & district nurses also attached to GP surgeries.	Provide pulmonary rehabilitation.		

**Table 2:** Participant description

**Participants interviewed**	**Age group**	**Number of prescribed medications**	**In paid employment**
Male with comorbidities (Ischaemic Heart Disease, stroke, arthritis) & wife (carer)	80 plus	> 3	Retired
Male with comorbidities (emphysema, arthritis) & wife (carer)	80 plus	> 3	Retired
Male with comorbidities (Chronic Obstructive Pulmonary Disease, Ischaemic Heart Disease, stroke) & wife (carer)	80 plus	> 3	Retired
Female with comorbidities (stroke, emphysema, Ischaemic Heart Disease) & husband (carer) (Ischaemic Heart Disease)	80 plus	> 3	Retired
Carer (wife) of man with comorbidities (emphysema, heart failure)	80 plus	> 3	Retired
Female with comorbidities (Parkinson’s Disease, osteoporosis, hypertension) & husband (carer) (Ischaemic Heart Disease)	80 plus	> 3	Retired
Female with comorbidities (asthma, heart failure, osteoporosis)	75-79	> 3	Retired
Female with comorbidities (Chronic Obstructive Pulmonary Disease, osteoarthritis, osteoporosis) and husband (carer) (Ischaemic Heart Disease)	75-79	> 3	Retired
Female with emphysema and hypertension	70-74	> 3	Retired
Male with comorbidities (diabetes type 2, Chronic Obstructive Pulmonary Disease, Ischaemic Heart Disease, arthritis) & wife (carer)	70-74	> 3	Retired
Female with comorbidities (emphysema, rheumatoid arthritis, atrial fibrillation) and husband (carer) (Ischaemic Heart Disease, depression)	70-74	> 3	Retired
Male with Parkinson’s Disease & wife (carer)	60-64	> 3	Retired
Female with Parkinson’s Disease	60-64	> 3	Retired
Male with diabetes type 1	60-64	> 3	Retired
Male with diabetes type 2	55-59	> 3	Yes
Male with diabetes type 2	55-59	> 3	No
Female with diabetes type 2	55-59	> 3	No
Female with Parkinson’s Disease	50-54	> 3	No
Male with diabetes type 1	50-54	2	Yes
Female with multiple sclerosis	40-44	0	No
Male with diabetes type 1	35-39	2	Yes

**Table 3:** Interview guide

**Topic area**	**Prompts**
**Tell me a little about yourself**	Your age?
	What occupation you are currently or were previously in?
	Your/the person you care for health problems?
	How long you/the person you care for have had these problems?
	What medications do you/they take?
**Tell me about the services you/the person you care for receives**	Nature and frequency of Health services – primary care, community services, hospital, rehabilitation, pharmacy, other.
	Social services – home care, day centres, other.
	Voluntary services – for example; meals on wheels, day centres.
**In your opinion how well do these services work together to coordinate the care?**	Can you give me some examples?
**How often do you have to tell the same information to several services?**	Can you give me some examples?
**How often do the services seem aware of what the others are doing for you/the person you care for?**	Can you give me some examples?
**Is there anything else you would like to tell me about the services you receive?**	

This paper reports on the qualitative findings from patients and carers in phase two of the study.

## Results

Patients and carers did not talk in terms of “coordination” or “integration” of services. Rather, their focus was on what features of a service reduced the workload of living with a long term condition and made life simpler.

### Services that made life simpler

Patients and carers receiving the community matron service had complex needs and were generally older than other respondents. They described how the community matron took over many aspects of the workload such as sorting out prescriptions, referring onto and obtaining rapid access to other services, being a mediator between all other services, and providing a sense of security which reduced anxiety.

…she (CM) does my blood pressure, sounds my chest, any worries, just no worries at all, I mean…*It’s general enquires, i.e., there’s no cure but we’ll give you as much relief as we can and that’s…*Yes. That’s it basically isn’t it, yes. *So I mean she’s* (CM) *quite good…*

“Alice” (emphysema) and “Donald” (carer)

There was also some evidence that the community matron (CM) service was reducing patient visits to the GP.

I’ve never requested to see a doctor, I just usually ring up “can I make an appointment to see (CM)?” *Well they should all be working together.* And she’s (CM) always been fantastic and if she’s felt that she would like me to see a doctor, “I’ll get a doctor in to see you”,

“Stan” (emphysema, arthritis) and “Mary” (carer)

The respiratory services were also spoken of in positive terms by patients. Again, these respondents were from the older age and of importance to them was ease of access to the service, particularly the nurse consultant (NC).

(NC) *… changed two of my sprays … Which made a big difference but I think the main thing is that I had always got antibiotics and steroids here so I know that I can start on them if I feel bad, I haven’t got to wait for an appointment and I ring* (NC) *up and* (NC) *comes down.*

“Lily” (Chronic Obstructive Pulmonary Disease, osteoarthritis, osteoporosis)

The neurological service was also well evaluated by service users who ranged in ages and were living with either Parkinson’s disease or multiple sclerosis. For them, what was important was the comprehensiveness of a service which included rehabilitation and social care support, plus being able to self-refer back to the service as required.

*They* (Neurological service) *make you feel looked after and considered, you know, they make you feel sort of as if they’re caring about you, and that’s obviously clearly just the way the system works, the way it does dovetail together I think really, it made you feel like you weren’t being fobbed off or anything like that, you know, just basically you felt looked after really … you’ve always got that security haven’t you, you feel like you could sort of just give them a ring…that’s a nice feeling you know.*

“Nicola” (multiple sclerosis)

### Issues with services

Services that were perceived to be personalised, gave a sense of being cared for, and provided a sense of security were described positively by respondents. However, the data also indicated that a number of service users felt there was a lack of coordination between the different aspects of the services they were receiving. Examples included blood test results not being transferred between services, and the need to repeatedly give their health history to different clinicians. Some respondents with more than one condition also described issues around being “separated” into diseases within the clinic/practice-based clinic.

*I go down to* (nurse practitioner running the GP based clinic) *and she says ‘Oh, only one thing this time, we’re only here for so-and-so’, and I’ve got a list…*

“Fred” (Diabetes type 2, bladder cancer, hypertension, atrial fibrillation)

For Fred and his wife this meant that they would have to make a number of separate appointments to address all his needs and making the appointment was equally challenging.

… we always have problems getting through to the surgery, then getting an appointment. It seems that if you can go online, but I don’t like using a computer… I don’t want computers.

### Hassles of living with a long-term condition

The biographical discussion at the beginning of each interview suggested that patients and carers were preoccupied with the “hassle” of living with a long-term condition with narratives focused on how services could reduce it. Within this paper we use Lazarus’ definition of a hassle being “*experiences and conditions of daily living that have been appraised as salient and harmful or threatening to the endorser’s well-being”* (p376) [35]. Included within these hassles was dealing with the consequences of fatigue, immobility or the difficulties in managing cumbersome equipment.

It’s taking the oxygen everywhere, this is the biggest thing probably. It’s okay taking bottles but you’ve got that big machine so you’ve got to lift that in and out the car. As you get older it’s, it doesn’t get easier does it?…we’ve given up on holidays now really, haven’t we?

“Donald” (carer)

Patients and carers’ descriptions of the challenges they faced on a daily basis could largely be linked to the consequences of the condition they were living with. However, irrespective of the age or predominant long-term condition of each respondent there appeared to be a common hassle which was raised by respondents themselves, often during the closing stage of the interview where they were invited to say anything else about the services they received not already covered within the interview.

### The repeat prescription – a recurring hassle

Even for those patients whose condition had minimal impact on their daily life, there was a recurring hassle that the majority of respondents reported; the repeat prescription. Liam was a younger man with type 1 diabetes mellitus. While he was generally positive about his experience of services, he also described his issues with managing his prescription.

…I find it a little bit frustrating at times because it’s all repeat stuff and then occasionally things won’t be in and I have to chase them…I get frustrated with on occasions if I’m running low on, whatever it is, needles or insulin, and I’m chasing and they say “Oh, won’t be in till Monday” …so that’s probably my only real, sort of, bugbear.

“Liam” (Diabetes type 1)

While the pharmacy not having stock available was commonly reported, the main problem appeared to be the amount of time not only spent on returning to the pharmacy once items came into stock, but also the extra time it took to manage prescribed medicine that was not synchronised.

I’ve got two pages of repeat prescriptions and goodness knows what, and they’re not, they don’t all fall at the same time, you know, so I spend an awful lot of time…

“Kath” (Emphysema)

Many patients such as Kath managed this issue covertly rather than asking for a review of her medication.

*…sometimes I know that I’ve got perhaps three or four things to get and there’s another one which I know I’ll have to in a week’s time, so I’ll say to the receptionist “look I know this isn’t due yet but to save me” … But you couldn’t do it with allof the prescriptions, you know, just the odd one.*

“Kath” (Emphysema)

On questioning, none of the participants we interviewed remembered receiving a medication review from their pharmacy. There was also evidence that they did not see the pharmacist as having a significant role on medicines management.

*…there’s forty things on his repeat prescription and my three as well…****And do you have to order them once a month?…****No, it doesn’t work out, they never all run out at the same time…****And has the chemist *(pharmacist) *said “oh let’s try and get this all sorted out for you”?****I don’t see how they could because some are for two months, some are for a month, some, you know?*

“Mary” (carer)

***And does the chemist offer you a medicines review?****Doctor does that doesn’t he? No, the doctor, looks through my tablets whenever they do, you know.*

“Sydney” (Diabetes type 2)

Alex and his wife described the effort required in making the repeat prescription more manageable.

The prescription. That’s just the sort of thing that you could really do without when you’ve got this condition… …I wrote clearly on my request, ‘Please can I have two months supply of L-Dopa’, because it does appear to help my symptoms and it would put it in synchronisation with the rest…I’ve got a maths degree…but when I asked the doctor for two months when she first prescribed it, she said ‘Oh, okay, I’ll give you two months, that’s 90 tablets’, and this is where the illness is annoying, because I was still able to work out that a month being roughly 30 days, 3 tablets a day, that’s only one month, and I sort of said ‘That’s only one month’. She said ‘No, no, that’s two months’, and I panicked a bit and I didn’t follow the argument through that it should have been 180.

“Alex” (Parkinson’s disease)

In one extreme case, Jeff with heart disease cared for his wife Mabel with rheumatoid arthritis, emphysema, atrial fibrillation and inflammatory bowel disease. They were both on multiple medications and were registered with different practices. Jeff described how he took the repeat requests to each surgery, returning two days later to pick them up and deliver to the pharmacy. When collecting the prescriptions he was frequently told by the pharmacist that he cannot dispense certain drugs for Mabel because of interaction alerts, with Jeff being asked to return to Mabel’s GP to query this. As for a number of other respondents, managing the repeat prescription proved to be one of the most demanding tasks on a week-by-week basis. This example illustrates the complexity of medication management coupled with a divided system between prescriber and dispenser that left the carer feeling unsupported.

The majority of patients we interviewed were on an established drug regimen (Table 2), but prescriptions only lasted 28 days. Although many respondents reported difficulties with the repeat prescription, there were some notable exceptions.

### Seamless repeat prescription systems

Different models of prescribing and dispensing repeat prescriptions were described. There were examples of the community pharmacy providing a service which removed any effort on the patient’s part in managing the repeat prescription, with the pharmacy ordering, dispensing and delivering the medicines to the patient’s house.

Despite having co-morbidities and complex needs, patients and carers receiving the community matron service did not report issues around their repeat prescriptions. Their narratives included accounts of the community matron organising seamless prescribing, medication review and delivery of the prescription that negated this particular hassle of living with chronic conditions.

We hadn’t appreciated the depth of the Community Matron’s, sort of, the sweep of her thing… she can call in more things than we thought, we thought she was just coming round to check on how we were, but apparently she can control quite a few things like our prescriptions.

“Arthur” (carer)

However, for many the time involved in submitting prescription requests in person, picking up the script, delivering to the chemist and then returning a few days later only to find that a number of items were out of stock proved to be the final irritation in negotiating long term conditions care delivery. Problems were exacerbated when the prescription only lasted 28 days meaning that the cycle of ordering, picking up, delivering and picking up was repeated more frequently.

## Discussion

The aim of this study was to explore patient experience of long-term conditions services which had elements of integration and were aiming to provide a seamless service. Our interview guide (Table 3) allowed participants to raise any examples of where care felt fragmented. The results suggest that fragmentation manifested itself in patient experience as labour-intensive challenges in the management of their condition, such as accessing GP appointments, but most predominantly in managing their repeat prescription. Corbin & Strauss [36] describe trajectory work as the activities a person does to manage their chronic illness. In this study emblematic of “trajectory” work was the effort required to ensure prescribed medication was available in the home. It appeared that this work was increased if repeat prescriptions were required every month rather than a longer period. The majority of patients and carers interviewed found managing repeat prescriptions a time consuming task, causing disruption to life. Reasons for this included having to make several journeys per prescription including the initial request and subsequent collections. Lack of synchrony was reported with medications finishing at different times; this in turn increased the number of visits to the surgery.

For the relatively few patients’ who did not report any difficulties with their repeat prescriptions, their repeat prescription requests to the GP were linked directly to the pharmacy, from where patients’ could collect their medication, or it was delivered to their home address. Similarly, patients receiving services where the repeat prescription was organised on their behalf, such as the community matron service, did not report any issues arising from their repeat medication.

**Figure 1:**
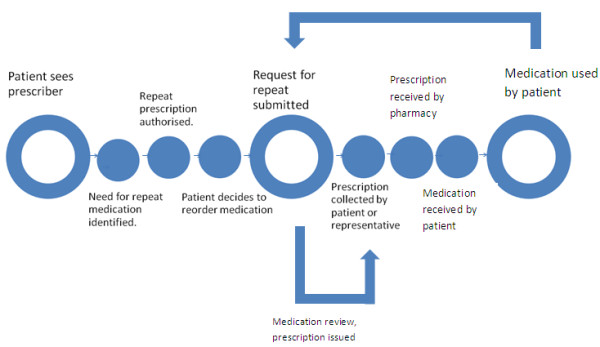
Repeat prescription ideal route map.

**Figure 2:**
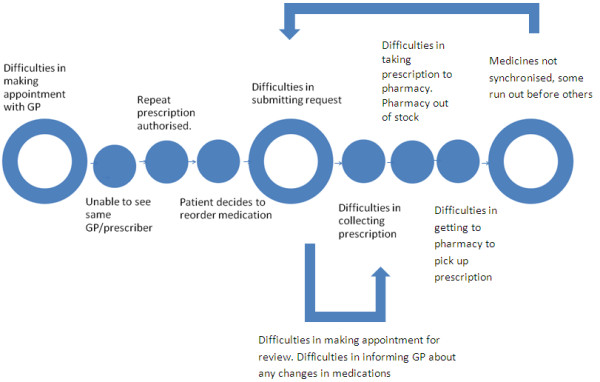
Recurring hassles of the repeat prescription.

However, in reality many patients and carers found themselves significantly deviated from the clear route map for repeat prescriptions as presented in the Good Practice Guide to Quality Repeat Prescribing [14] (Figure 1), with their actual experience a more difficult route (Figure 2).

There is little previous research on patient experience of obtaining prescriptions. A review of the evidence around older people and medicine management highlighted a number of studies that suggested reasons for poor medication adherence in older people including; health beliefs, therapy related factors and some system and health care team related factors such as the packaging and labelling of medicines [37]. However, a national survey and interview study within England reported that family carers experienced difficulties in maintaining a continuous supply of medications for the person they cared for [38]. These difficulties included the increased burden of making repeated visits to the GP surgery to sort out any problems with the repeat prescription; however no link was made to the length of prescription. A report by AT Kearney commented on patient expectations of pharmacies [39]. A significant proportion of patients stated that pharmacy opening hours did not suit their lifestyle needs, and also that waiting times for prescriptions were too long [39]. Our findings suggest that many of the patients interviewed were dissatisfied with the length of their repeat prescription. Much literature exists about the cost, length and wastage of medicines. The National Health Service spends £8 billion a year on prescription drugs in primary care in England. Expenditure on primary care drugs has increased by 60 per cent over the last decade, and the number of items dispensed has increased by 55 per cent [8,23]. It is estimated that up to £300 m is wasted (medicines dispensed but not consumed) in NHS primary and community care prescription drugs in England annually, and that £150 m of the medicines waste is avoidable [23]. The National Audit Office published a report in 2007 criticising the prescribing of doctors and levels of pharmaceutical waste [8], estimating that cost of waste varies from 1% to 10% of total spending on medicines [40,41]. As a response to the rising cost of medicine waste, Primary Care Trusts, with the encouragement of the National Prescribing Centre, introduced initiatives to reduce the number of dosage units to a 28-day supply [8,41,42]. The effect of these initiatives is clear with a 2008 study indicating that the average length of a prescription was 40 days [40], whereas a recent study looking at trends in prescribing data from 1998 – 2009 in England for 11 medicines showed a significant drop in doses per prescription. However, this equated to 35 million more items being dispensed in 2009 compared to 1998. It is estimated that this cost the NHS an extra £150 m a year in dispensing fees [41].

There is evidence of patient organisations lobbying for changes to prescription length. The Patients Association argued that the majority of patients are unhappy with a 28-day supply of medicines; one reason for this is the rise in cost for the patient. Patients with long-term conditions need multiple prescriptions, paying prescription charges more often [42]. In an open letter to the Department of Health [43], the Addison’s Disease Support Group stated that reducing prescriptions to 28 days is disempowering for patients, causing anxiety when running low, particularly over weekends. It also limits the freedom to travel.

There is limited previous research exploring the effect of prescription length. A UK survey [44] looking at trends in the prescribing of thyroid hormone medication showed that prescription duration over the last 10 years has reduced from 60 to 45 days on average. The survey showed great dissatisfaction with the 28-day prescription (59% dissatisfied overall, 13% satisfied). Fewer than half of those given a 28-day prescription had asked their GP for a greater supply. Of concern is that 17% admitted to missed medication, 6% having gone without tablets on more than one occasion owing to lack of dispensed medication [44]. However, some patients thought obtaining repeat prescriptions would be easier to remember if all the medicines were given for 28 days, and would improve synchrony. It allows for production of a 28-day “blister pack” which makes it easier for patients to remember medication [44].

The National Prescribing Centre in 2008 released further information regarding the 28-day policy, advising that longer prescription periods may be suitable for those patients who suffer with long-term conditions and can safely manage their medication [18]. Despite this advice, prescribers routinely write prescriptions for 28 days [18,41,44]. A recent report commissioned by the Department of Health [11] made no explicit mention of the impact of prescription length, but focused on the link between improved repeat prescribing, improved health outcomes and reduced dispensing of medicines that were no longer required. However, while the report recommended improved communication with patients about prescribing decisions, it did not identify the need for improving mechanisms for feedback from patients about their experiences of repeat prescription services.

There are several advantages and disadvantages to a 28-day prescribing policy. However there is very little previous evidence evaluating how this change in prescribing behaviour has impacted upon patients. Up to now, patient experience has played little part in the wastage versus prescription cost debate, but with an increasing focus on improving quality of care delivered, and the vast changes occurring within the NHS, it is vital to assess all components of quality [45]. Evaluating the needs and preferences of service users is an important aspect in reviewing the quality of service delivered [46,47]. Patient satisfaction serves as an essential determinant of viability and sustainability of health care services [48], and stockpiling of medications by patients may be a strategy to prevent facing the rigours of organising a monthly prescription when situations are particularly difficult. While it is recommended that patient satisfaction should be taken into account when designing a repeat prescription service [14], the effect of flaws within the system upon patient adherence has not been previously identified. Aspects of the current system within England are unlikely to change in the near future as the current reimbursement schemes for community pharmacies in England (fee per dispensed item) makes it unlikely that lengthening repeat prescriptions would be encouraged by pharmacists. Nevertheless, it has been estimated that even moving 50% of prescriptions from 28 days to 3 months in the 4 main chronic diseases would reduce items dispensed by over 40 million, significantly reducing system costs [39].

Pharmacies in England are undergoing further change, as the Electronic Prescription Service release 2 (EPS2) is due to be introduced [49]. This system will enable prescribers to digitally sign and electronically transfer the prescription to a national database, ‘The Spine’. The prescriptions can then be downloaded and dispensed by a pharmacy chosen by the patient. It is hoped that this will optimise pharmacy processes, and improve organization; for example being able to download prescriptions at the start of business, and dispense prior to GP practices opening. This would allow a smooth flow of dispensing throughout the day [49]. Evaluation of how this will impact on the patient experience will be of interest once implemented.

The study we report has limitations in terms of being confined to patients in just two primary care trusts. We were also dependent on participants being recruited through the actual services and have no information about patients and carers who did not wish to take part in the study. The focus of the study was not specifically on repeat prescriptions and in the first phase of the study we had not collected data from health professionals on their perspective of repeat prescriptions. However, as this finding emerged inductively from patient and carer interviews we are confident of the validity of our findings, and that interviews were conducted until data saturation had occurred with no new themes arising [50].

## Conclusion

While this study is limited in terms of the number of research sites, it highlights a previously under-reported area in an increasingly significant area of health spending. With an ageing population and associated morbidities, a system change in repeat prescribing which would not only reduce costs but also improve patient experience, appears overdue. There seems to be a clear case for increasing routine repeat prescriptions to a longer duration in terms of reducing costs and improving patient satisfaction, however further research is required to explore how this would affect patient and carer burden, and health outcomes.

## Competing interests

The authors declare that they have no competing interests.

## Authors’ contributions

PW conceived of the study and design, participated in data collection and analysis, and helped draft the manuscript. NK participated in data analysis and interpretation, and helped draft the manuscript. EM helped with data collection, analysis and critically revising the manuscript. All authors read and approved the final manuscript.

## Pre-publication history

The pre-publication history for this paper can be accessed here:

http://www.biomedcentral.com/1472-6963/13/192/prepub
